# Supplementary material for: Chromothripsis during telomere crisis is independent of NHEJ, and consistent with a replicative origin
Source: Genome Res. 2019 May;29(5):737–49. doi: 10.1101/gr.240705.118 (PMC6499312; doi:10.1101/gr.240705.118)
Supplement: Supplemental Material [file supp_gr.240705.118_Supplemental_file_1.zip › contigs/annotated_contigs/DB81/contig.2.DB81_length_250_mean_cov_6.6.docx]

**DB81_length_250_mean_cov_6.6**

```
CAGACATTAGTCTGACCTCCAGGGCCACCTGCACTCCCCAGCCAATCAGCCACTACATGAACAATGCCCTGTAACACCCTAAACTTGGG
 >chr3:51722552-51722716 - E=1e-87
AGAGGAGGCTCAAACCATTTTACAAAGACCCTCCCCCTAAAAACTCCCTGAAATCAGAGACCATGGGGAAGGGCA|GGAAGT|GGCAGG
                                                                                  >chr3
ATGGACACGGCGGGTAGCCAGCCAGATGGACAGAATGGTGGTGTAGGGGAAGGTGGCTGTCACCACATGGCTGG:51718552-51718632 - E=6e-37
```
